# Evaluating the Efficacy of Sequential Microimmunotherapy Medicine on Early and Established Postmenopausal Osteoporosis Rat Model

**DOI:** 10.1155/joos/7645840

**Published:** 2026-07-29

**Authors:** Maria Del Mar Ferrà-Cañellas, Laura Garcia-Sureda, Joana Maria Ramis, Marta Monjo

**Affiliations:** ^1^ Group of Cell Therapy and Tissue Engineering, Research Institute on Health Sciences (IUNICS), University of the Balearic Islands, Palma, Spain, uib.es; ^2^ Preclinical Research Department, Labo’life España, Consell, Spain; ^3^ Health Research Institute of the Balearic Islands (IdISBa), Palma, Spain

**Keywords:** bone resorption, C-terminal telopeptide fragment of Type I collagen, mechanical test, microimmunotherapy, osteoporosis, ovariectomized rat model

## Abstract

Postmenopausal osteoporosis (OP) is characterized by an imbalance between bone formation and resorption due to declining estrogen levels, leading to progressive bone loss and increased fracture risk. This study aimed to evaluate the effects of the sequential microimmunotherapy medicine 2LOSTEO‐N (MIM‐seq) in two rat models of OP. Treatment began either 3 weeks (early‐onset OP) or 15 weeks (established OP) after ovariectomy (OVX) and was administered orally for 9 weeks. The impact of MIM‐seq was assessed through (i) body composition analysis, (ii) plasma bone turnover markers—CTX‐I, alkaline phosphatase (ALP), osteoprotegerin (OPG), and RANKL—and (iii) bone quality by mechanical testing and micro‐computed tomography (micro‐CT) of trabecular bone morphology. In early‐onset OP, MIM‐seq improved vertebral strength, reduced CTX‐I levels, and increased the OPG/RANKL ratio, suggesting a decrease in bone resorption. In established OP, MIM‐seq also significantly reduced CTX‐I levels. However, micro‐CT analysis did not show significant changes in bone microarchitecture in either model. Overall, this study highlights the biological response to MIM‐seq in experimental OP, indicating a modulatory effect on bone turnover markers and mechanical strength in early disease stages. Although no significant microstructural improvements were detected, these findings suggest potential therapeutic benefits of MIM‐seq in bone health management.

**Mini Abstract**
• This study assessed the effects of MIM‐seq in early and established postmenopausal osteoporosis (OP) rat models. MIM‐seq reduced bone resorption biomarkers and improved vertebral strength in early‐stage OP. Although no microarchitectural changes were observed, results suggest treatment potential to modulate bone turnover and contribute to OP management.

## 1. Introduction

Osteoporosis (OP) is a bone disease characterized by low bone mineral density and microstructural deterioration, leading to bone fragility and fractures [1]. It results from abnormal bone remodeling, decreasing bone strength, and increasing susceptibility to bone fractures [2]. The immune system plays a critical regulatory role in OP [3], a relationship known as “osteoimmunology,” where signaling factors and cytokines influence bone remodeling [4, 5]. The coupling between the processes involved in OP needs an intimate form of crosstalk between cells and systemic and local factors. Hence, different studies highlight the need to balance immune responses to promote bone formation and manage OP [6]. OP pathogenesis involves systemic inflammation and immune response dysregulation [7]. Evidence links OP to inflammation‐associated bone disorders, like rheumatoid arthritis or periodontal diseases [8, 9], both involving increased osteoclastic activity, collagen breakdown, and inflammatory pathways contributing to bone loss [10].

Current OP management aims to enhance bone density by reducing bone resorption or stimulating formation. Approved drugs include estrogen agonists/antagonists, bisphosphonates, receptor activator of nuclear factor kappa‐Β ligand (RANKL) inhibitors, and sclerostin inhibitors [11]. However, these therapies could be associated with potential side effects, such as kidney issues, gastrointestinal problems [12], and increased risks of vascular diseases or cancer [13] although generally, their benefits outweigh the risks [11, 14]. Osteoinductive growth factors like bone morphogenetic protein‐2 (BMP‐2) require high doses, leading to complications such as ectopic bone formation and osteoclast‐mediated resorption [15].

Innovative strategies are needed to improve treatment efficacy and safety, including dose reduction and optimized delivery systems [16]. With this aim, microimmunotherapy (MI) is a immunotherapy based on the administration of reduced doses of immunomodulatory molecules [17]. Within this approach, micro‐immunotherapy medicines (MIMS) use low dose (LD) and ultralow dose (ULD) of immunomediators to modulate the immune response [18] and support homeostatic balance [19]. MI has demonstrated safety, biological activity, and potential benefits in acute and chronic diseases [19, 20]. The sequential MI tested in this study 2LOSTEO‐N (hereafter referred as MIM‐seq) has shown positive anti‐inflammatory effects and collagen metabolism restoration in an in vitro periodontitis model [21].

The ovariectomized (OVX) rat model, widely used for postmenopausal OP research, simulates estrogen deficiency–induced bone loss [22, 23]. This model is cost‐effective, with skeletal effects manifesting within a month [22, 24]. This study included two OP rat models; an early‐onset model to assess MIM‐seq as a preventive treatment and an advanced OP model to evaluate its therapeutic efficacy. The early‐onset OP model induces conditions predisposing rats to OP without fully manifesting the disease, while the second model was used to assess the efficacy of MIM‐seq in reversing or mitigating existing OP.

Therefore, in the present study, MIM‐seq was daily oral administered for 9 weeks, in the two different OP models. The effect of MIM‐seq treatment on (i) body composition, (ii) plasma biochemical markers of bone turnover and collagen breakdown, and (iii) the bone quality properties through biomechanical testing and micro‐computed tomography (micro‐CT) analysis of trabecular bone morphology have been checked. To our knowledge, this is the first in vivo study evaluating the effects of 2LOSTEO‐N (MIM‐seq) in an OP model.

## 2. Materials and Methods

### 2.1. Animal Model and Ethical Approval

The experimental protocol of the study was approved by the Ethical Committee of Animal Experimentation (CEEA‐UIB) and the Direcció General d’Agricultura *i* Ramaderia (2019/16/AEXP). The experimental animals were 34 female Wistar rats (12 weeks, 200–250 g) (Envigo, Spain). OVX procedures involved abdominal incision and bilateral ovarian excision. Envigo housed rats for 1 week postoperatively in the early‐onset model and 10 weeks in the established model. Before experimentation, all rats were acclimated and trained for treatment intake. Animals were kept under constant conditions (20°C–24°C, 12 h light/dark), with ad libitum food (LASQCdiet Rod18‐H; calcium 10 g/kg, phosphorus 6.5 g/kg, and vitamin D3 1.200 IE/kg) and deionized water. The 24‐week‐old OVX‐established OP rats were randomized into treated with the MIM‐seq (*n = *9) and treated with the vehicle (*n = *9). The remaining 15‐week‐old OVX early‐onset OP rats were randomized into treated with the MIM‐seq (*n = *8) and treated with the vehicle (*n = *8).

### 2.2. Tested MIM

The tested MIM‐seq (2LOSTEO‐N) contains 10 capsules of sucrose–lactose pillules impregnated with active substances via serial kinetic process (SKP) [25]. MIMs are typically administered sublingually in humans, but in vivo preclinical research, they can be administered either orally or through gavage [19, 26].

The ingredients of the composition of this MIM‐seq are presented in “centesimal Hahnemannian dilutions” (CH). The CH dilutions represent the number of dilutions of the original stock for each component by a factor of 1/100 [25]. While the composition of each of the 10 capsules of the tested MIM‐seq has not been revealed due to confidentiality issues related to intellectual property, the complete list of ingredients include recombinant human (rh) interleukin (IL)‐1β (17 CH), rh‐IL‐6 (17 CH), rh‐IL‐11 (15 CH), rh‐insulin growth factor (IGF)‐1 (9 CH), rh‐granulocyte macrophage colony‐stimulating factor (GM‐CSF) (15 CH), rh‐transforming growth factor‐beta (TGF‐β) (5 CH), rh‐tumor necrosis factor‐alpha (TNF‐α) (17 CH), rh‐bone morphogenetic protein (BMP)‐2 (5 CH), and rh‐BMP‐4 (5 CH), nucleic acid based‐molecules such as deoxyribonucleic acid (DNA) (5 CH), ribonucleic acid (RNA) (5 CH), and specific nucleic acids (SNAs) such as SNA‐OSTEOa‐02 (18 CH) and SNA‐OSTEOb‐02 (18 CH), targeting human leukocyte antigen (HLA) I and II, respectively, and the fluoride derivative natrum silico fluoricum (Na_2_SiF_6_) (3 CH). The vehicle consisted of sucrose–lactose pillules impregnated with an ethanolic solution without any active substance, produced as experimental control for preclinical research [27].

### 2.3. Oral Treatment and Experimental Procedures

Daily oral administration of MIM‐seq lasted 9 weeks. Administration respected the MIM sequence which followed the order of the blister, from capsules 1 to 10 (one capsule/day). At the end of the first blister, on the 11th day, the sequence was restarted with a new blister, until the end of the treatment. In total, six complete sequences were administrated. The vehicle solution consisted only of the sucrose–lactose pillules with no active substances. The study was blinded during experiment, outcome assessment, and the data analysis.

**FIGURE 1:**
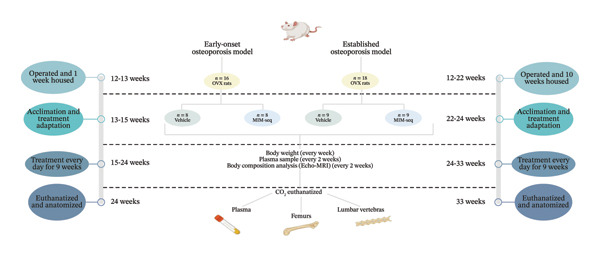
Schematic timeline of the experimental procedure. For the early‐onset osteoporosis (OP) model, 16 female Wistar rats of 12 weeks were bilaterally ovariectomized (OVX) and housed for 1 week. At 13 weeks, rats were acclimated and adapted to the treatment. At 15 weeks, daily oral treatment began, and it continued for 9 weeks. At 24 weeks, rats were euthanatized and dissected for the experimental procedures. For the established OP model, 18 female Wistar rats of 12 weeks were bilaterally OVX and housed for 10 weeks. At 22 weeks, rats were acclimated and adapted to the treatment. At 24 weeks, daily oral treatment with the sequential microimmunotherapy (MIM‐seq) of interest or the vehicle began for 9 weeks. At 33 weeks, rats were euthanatized and dissected for the experimental procedures. Figure created with BioRender.com.

Oral administration was done following the syringe‐feeding technique [28]. Before treatment, animals learned to drink from the syringe voluntarily and reliably, whereas drinking latency decreased after 1 week of adaptation. Then, treatments were given sequentially as indicated, giving voluntary oral administration. Capsules were dissolved in 1.5 mL of water (1 capsule dissolved in 1.5 mL of water/rat/day). The control group received vehicle in the same volume (1.5 mL). Treatment began in the 12^th^ week after surgery of OVX rats with established OP. Daily oral administration lasted for 9 weeks. A schematic timeline is shown in Figure 1.

Body weight was recorded weekly. Every 2 weeks, blood sample was drawn from the lateral saphenous vein for plasma collection, and body composition was analyzed via Echo MRI (EchoMRI, LLC, Houston, TX, USA). Plasma was separated by centrifugation (1500 x g, 15 min, RT) and stored at −80°C. Nine weeks posttreatment, animals were euthanized with CO_2_ (> 70%) in a carbon dioxide chamber, legs were fixed in 4% paraformaldehyde, and lumbar (L4) vertebrae were stored at −20°C.

### 2.4. Plasma Sample Collection

Blood samples were collected from the lateral saphenous vein every 2 weeks. After hair removal and glycerin application to aid blood collection, a 25‐gauge needle (Sarstedt, Nümbrecht, Germany) punctured the vein at a 90° angle at the most proximal visible site. Blood (300 μL) was collected into ethylenediaminetetraacetic acid (EDTA)–coated capillary blood collection tubes (Microvette CB 300 EDTA potassium, Sarstedt), mixed gently inversion to prevent hemolysis. Pressure was applied to stop bleeding. Plasma was separated by centrifugation (1500 × g, 15 min RT), within 30 min and stored at −80°C, except for alkaline phosphatase (ALP) activity, which was determined from fresh plasma.

### 2.5. Plasma Biochemical Parameters

ALP activity was determined in fresh plasma after 9 weeks using p‐nitrophenyl phosphate (pNPP; Sigma, Saint Louis, Missouri, USA), with calf intestinal alkaline phosphatase (CIAP; Promega, Madison, USA) for calibration. Bone turnover markers, osteoprotegerin (OPG; Abbexa Ltd, Cambridge, United Kingdom, Catalog No.: abx255875), RANKL (Abbexa Ltd, Catalog No.: abx257583), and C‐terminal telopeptide fragment of Type I collagen (CTX‐I; Abbexa Ltd, Catalog No.: abx155410) were quantified via enzyme‐linked immunosorbent assay (ELISA) assay following the manufacturer’s instructions.

### 2.6. Mechanical Testing

L4 vertebrae were isolated and subjected to compression testing using a universal testing machine (Zwick/Z100, Ulm, Germany). Load was applied at 1‐mm/min RT until fracture. Maximum load applied (N) was recorded via Instron software (INSTRON series IX Automated Materials Tester, Version 8.04.00).

### 2.7. 3D Femur Trabecular Microarchitecture Analysis by Micro‐CT

Right femurs fixed in 4% paraformaldehyde were analyzed via micro‐CT. Trabecular structure of the right femur metaphysis was assessed using micro‐CT scanner (model V Tome *X* s 240 de GE Sensing & Inspections Technologies, Wunstorf, Germany), with X‐ray tube voltage of 160 kV, current of 160 μA, and a 2 × 0.1‐mm copper filter. Scans had a 360° angular rotation, 0.2° angular increment, and an 18.5‐*μ*m voxel size. A 3D morphometric analysis was conducted using the CT‐analyser (CTan) software (Skyscan, Aartselaar, Belgium). Trabecular bone was analyzed in the proximal metaphysis region, starting just distal to the metaphysis and continuing distally conforming the region of interest (ROI). ROI was selected, taking care that no part of the cortical bone was included and that the gap between the inner cortical bone and the contour was constant and the same for all samples. Care was also taken that the growth plate was not included in the ROI. Such a contour was drawn every 15^th^ slice, defining the ROI for the evaluation in every sample. The lower gray threshold was set at 130 and the upper gray threshold at 250 for all the evaluations.

The 3D morphometric parameters obtained were bone volume/total volume (BV/TV, ratio of the segmented bone volume to the total volume of the ROI), bone surface density (BS/TV, ratio of the segmented bone surface to the VOI), trabecular thickness (Tb. Th, mean thickness of trabeculae), trabecular separation (Tb. Sp, mean distance between trabeculae), trabecular number (Tb. N, measure of the average number of trabeculae per unit length), trabecular pattern factor (Tb. Pf, measure the relative convexity or concavity of the total bone surface), structure model index (SMI), change in surface curvature), and the degree of anisotropy (DA, degree of directional organization).

### 2.8. Statistics

Data are mean values ± standard error of the mean (SEM). Normality was assessed via the Shapiro–Wilk test. Groups differences were assessed by the unpaired *t*‐test or Mann–Whitney test (*p* < 0.05). SPSS program (Version 17.0, SPSS Inc, Chicago, IL, USA) and GraphPad Prism (Version 7, La Jolla, CA, USA) were used.

## 3. Results

### 3.1. Early‐Onset OP Model

**FIGURE 2:**
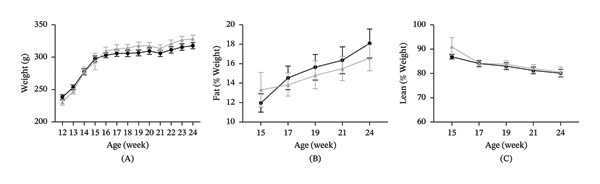
Temporal changes of body composition in ovariectomized (OVX) rats with early‐onset osteoporosis (OP). Body weight (A), fat mass (B), and lean mass (C) were determined by Echo MRITM body composition analyzer. Groups are OVX animals (OVX) which were administered the vehicle () or MIM‐seq (). Results represent the mean ± standard error of the mean (SEM), with *n = *8 for each group. Results were compared for statistical significance by unpaired *t*‐test analysis. No significant differences were found.

This first part of the study evaluated the effect of MIM‐seq administration on early‐stage bone loss in OVX rats. Three weeks postsurgery, OVX rats received daily oral MIM‐seq treatment or vehicle control for 9 weeks (Figure 1). As shown in Figure 2A, body weights gradually increased the first 4 weeks and then stabilized, with no significant differences between groups. Similarly, fat mass increased (Figure 2B) and lean mass decreased (Figure 2C), without statistical significance.

**FIGURE 3:**
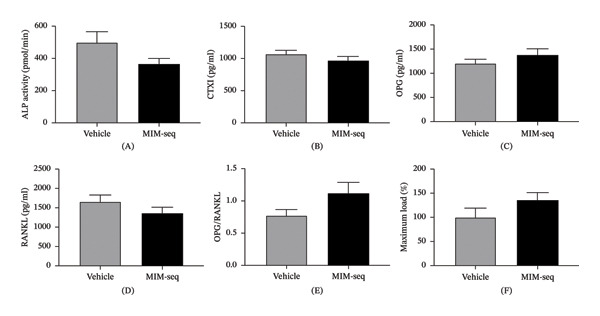
Plasma levels of bone turnover biomarkers determined by enzyme‐linked immunosorbent assay (ELISA) and assessment of mechanical properties in rats with early‐onset osteoporosis (OP) after 9 weeks of treatment. (A) Plasma alkaline phosphatase (ALP) activity was used as a bone turnover marker. (B) Plasma levels of the C‐terminal telopeptide fragment of Type I collagen (CTX‐I) were used as a marker of bone resorption. (C) Osteoprotegerin (OPG). (D) Receptor activator ligand of nuclear factor kappa‐B (RANKL). (E) OPG/RANKL protein ratio. (F) Maximum load values of the fourth lumbar vertebral body (L4). Results represent the mean ± standard error of the mean (SEM), with *n = *8 for each group, and were compared by unpaired *t*‐test analysis. Results were compared for statistical significance by paired *t*‐test analysis. No significant differences were found.

Plasma ALP activity and CTX‐I plasma levels, as markers of bone turnover, increased in OP. Lower plasma ALP activity (−26.7%) was seen for the MIM‐seq compared with the vehicle although this reduction was not statistically significant (*p* = 0.152) (Figure 3A). Regarding plasma CTX‐I concentrations (Figure 3B), slightly lower concentrations (−15.0%) were found for the MIM‐seq‐treated group in comparison to the vehicle one although these differences did not reach statistical significance neither (*p* = 0.241).

In this study, we also evaluated the effect of sequential treatment in preventing estrogen‐deficient bone loss by modulating the release of RANKL and OPG. As shown in Figure 3C,D, the MIM‐seq group showed higher OPG plasma levels (+14.6%) and lower RANKL plasma levels (−17.9%) than the vehicle group although these differences did not reach statistical significance. The OPG/RANKL protein ratio (Figure 3E) showed an increase (+44.8%) in the MIM‐seq‐treated animals compared with the vehicle ones, without reaching significance (*p* = 0.083).

Finally, the biomechanical properties of the L4 vertebral body were evaluated using a compressive test (Figure 3F). Maximum load in the treatment group was 36.2% higher than the vehicle group although differences were not statistically significant (*p* = 0.157).

The trabecular bone microarchitecture of the right distal femur was measured and evaluated using micro‐CT analysis. Table 1 presents the values of the micro‐CT parameters affecting the trabecular microarchitecture for both the vehicle and MIM‐seq groups. No statistically significant differences were observed between the treatment and vehicle groups.

**TABLE 1:** Evaluation of bone microarchitecture as measures of bone quality of early‐onset osteoporosis (OP) rat model after 9 weeks of treatment. The 3D trabecular microarchitecture analysis of femur by micro‐CT in ovariectomized (OVX) rats after being given vehicle or treatment administration for 9 weeks.

Parameter	Vehicle	MIM‐seq	*p* value
BV/TV (%)	13.1 ± 0.6	13.1 ± 0.7	0.995
BS/TV (1/μm)	0.118 ± 0.004	0.115 ± 0.005	0.613
Tb. Th (μm)	3.92 ± 0.08	4.01 ± 0.09	0.474
Tb. Sp (μm)	43.4 ± 0.8	42.3 ± 1.5	0.520
Tb. N (1/μm)	0.033 ± 0.001	0.033 ± 0.002	0.725
Tb. Pf (1/μm)	0.128 ± 0.014	0.128 ± 0.011	0.995
SMI	1.55 ± 0.05	1.55 ± 0.06	0.988
DA	2.35 ± 0.66	3.34 ± 1.32	0.645

*Note:* The parameters studied are percent bone volume/total volume (BV/TV), bone surface density (BS/TV), trabecular thickness (Tb. Th), trabecular separation (Tb. Sp), trabecular number (Tb. N), trabecular pattern factor (Tb. Pf), structure model index (SMI), and degree of anisotropy (DA). Results represent the mean ± SEM, with *n = *9 for each group. Results were compared for statistical significance by unpaired *t*‐test analysis. No significant differences were found.

### 3.2. Established OP Model

The second part of the study evaluated the effect of sequential MIM‐seq administration on reducing bone loss in the established OP model. Twelve weeks postsurgery, OVX rats received daily oral MIM‐seq treatment or vehicle control for 9 weeks (Figure 1).

**FIGURE 4:**
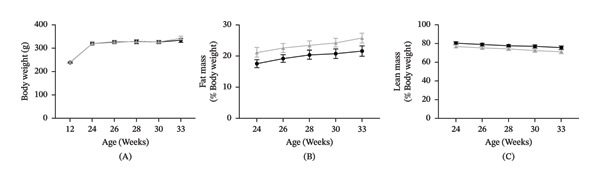
Temporal changes of body composition in ovariectomized (OVX) rats with established osteoporosis (OP). Body weight (A), fat mass (B), and lean mass (C) were determined. Groups are OVX animals which were given the vehicle () or MIM‐seq () treatment. Results represent the mean ± SEM, with *n = *9 for each group. Results were compared for statistical significance by unpaired *t*‐test analysis. No significant differences were found.

As shown in Figure 4A, at the beginning of housing weeks, the body weight increased gradually with age, as expected. However, during the 9‐week treatment period, the body weight was constant and nearly indistinguishable between groups. No significant differences between groups were observed in the fat mass percentage (Figure 4B) and lean mass (Figure 4C).

**FIGURE 5:**
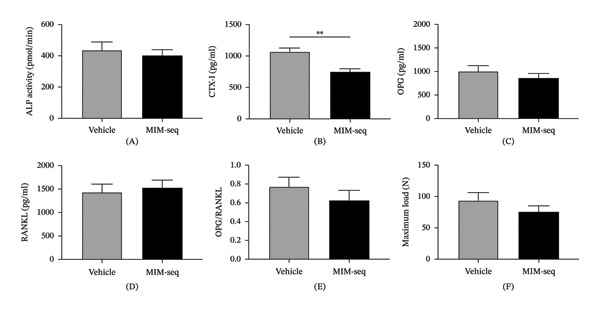
Plasma levels of bone turnover biomarkers determined by enzyme‐linked immunosorbent assay (ELISA) kits and assessment of mechanical properties in rats with established osteoporosis (OP) after 9 weeks of treatment. (A) Plasma alkaline phosphatase (ALP) activity was used as a bone turnover marker. (B) Plasma levels of the C‐terminal telopeptide fragment of Type I collagen (CTX‐I) were used as a marker of bone resorption. (C) Osteoprotegerin (OPG). (D) Receptor activator ligand of nuclear factor kappa‐B (RANKL). (E) OPG/RANKL protein ratio. (F) Maximum load values of the fourth lumbar vertebral body (L4). Results represent the mean ± standard error of the mean (SEM), with *n = *9 for each group, and were compared by unpaired *t*‐test analysis. Differences were considered statistically significant for *p* < 0.05, ^∗∗^represents *p* < 0.001 compared with vehicle.

Figure 5A shows the results of ALP as a marker of bone turnover, measured after 9 weeks of treatment. No significant difference was observed comparing the MIM‐seq and the vehicle groups at Week 33. However, a significant effect was seen for the bone resorption marker CTX‐I (Figure 5B), showing lower plasma CTX‐I concentrations (−30.0% *p* = 0.001) at the end of the experiment for the MIM‐seq group compared with the vehicle group. No significant difference was observed in the plasma levels of OPG (Figure 5C), RANKL (Figure 5D), the OPG/RANKL ratio (Figure 5E), and for the compressive test of the L4 vertebral body (Figure 5F).

Table 2 shows the values of the trabecular bone microarchitecture of the right distal femur by micro‐CT analysis. Differences between treatment and vehicle groups were not statistically different.

**TABLE 2:** Evaluation of bone microarchitecture as measures of bone quality of established osteoporosis (OP) rat model after 9 weeks of treatment. The 3D trabecular microarchitecture analysis of femur by micro‐CT in ovariectomized (OVX) rats after being given vehicle or treatment administration for 9 weeks.

Parameter	Vehicle	MIM‐seq	*p* value
BV/TV (%)	12.9 ± 0.4	13.9 ± 0.7	0.226
BS/TV (1/μm)	0.116 ± 0.003	0.120 ± 0.004	0.445
Tb. Th (μm)	3.93 ± 0.05	4.04 ± 0.13	0.435
Tb. Sp (μm)	42.0 ± 1.1	40.0 ± 2.3	0.440
Tb. N (1/μm)	0.033 ± 0.001	0.035 ± 0.001	0.331
Tb. Pf (1/μm)	0.152 ± 0.006	0.143 ± 0.011	0.506
SMI	1.62 ± 0.03	1.58 ± 0.04	0.470
DA	1.87 ± 0.08	1.67 ± 0.06	0.071

*Note:* The parameters studied are percent bone volume/total volume (BV/TV), bone surface density (BS/TV), trabecular thickness (Tb. Th), trabecular separation (Tb. Sp), trabecular number (Tb. N), trabecular pattern factor (Tb. Pf), structure model index (SMI), and degree of anisotropy (DA). Results represent the mean ± SEM, with *n* = 8 for each group. Results were compared for statistical significance by unpaired *t*‐test analysis. No significant differences were found.

## 4. Discussion

Postmenopausal OP exemplifies the interplay between immune, bone, and endocrine systems [6]. Osteoimmunology highlights the multiregulatory pathways connecting these systems [5]. The treatment evaluated, MIM‐seq, contains growth factors and cytokines targeting osteoclastogenesis (IL‐1β, IL‐6, TNF‐α, and IL‐11, GM‐CSF [20, 26, 27, 29], osteoblastogenesis (TGF‐β [30], IGF‐1 [31], BMP‐2, and BMP‐4 [30, 32, 33]), and inflammation modulation to rebalance bone turnover.

MIM has been investigated in other bone diseases, such as osteoarthritis and RA. Studies indicate that ULD MIM cytokines (including TNF‐α and IL‐1β), when combined with other immune factors, reduce RA inflammation in vivo [20, 26]. Additionally, LD cytokines and antibodies (IL‐4, IL‐10, and anti‐IL‐1 antibodies) enhanced treatment efficacy over disease‐modifying antirheumatic drugs (DMARDs) in RA patients [34]. In vitro, LD IL‐10 and anti‐IL‐1 antibodies modulated inflammatory pathways in chondrocytes, suggesting potential benefit for OP by inhibiting inflammation and bone resorption [35].

These results suggest that MIM‐seq may influence the immune system and bone remodeling, with further studies needed to elucidate its therapeutic potential in OP. Indeed, the tested MIM‐seq uses active substances to potentially target and act on inflammation, such as ULD IL‐1, IL‐6, TNFα, and substances affecting the extracellular matrix and bone remodeling, mainly LD of BMP‐2 and BMP‐4, ULD of IGF‐1 and GM‐CSF. In this context, the present study provides novel in vivo evidence supporting the potential of this sequential MI approach in OP.

Given these immune‐modulating effects, we assessed MIM‐seq in two OVX‐induced OP models to explore its potential impact on bone turnover and quality. The main difference between the two OP models is the timing of post‐OVX intervention: of three weeks in the early‐onset model versus 12 weeks in the established model. Bone response to OVX depends on age, bone type, site, and duration of OVX [36]. Moreover, there is a significant temporal correlation between bone loss and bone turnover in OVX rats, where the initial rapid phase of bone loss in OVX rats is coincident with the maximal increase in bone turnover, while at later times post‐OVX, both bone loss and bone turnover decrease [37]. Therefore, the present study examines OP models in growing rats, emphasizing temporal bone responses. The early‐onset model simulates conditions predisposing to OP, providing a platform to evaluate MIM‐seq preventively, while the established model evaluates its efficacy in advanced well‐established OP.

As reported in previous studies, OVX led to an increased body weight and fat deposition, compared with sham‐operated rats [24, 36]. This weight increase arose from a fattier deposition, as both the bone mass and lean mass index decreased after OVX, as previously described [38].

MIM‐seq was administered using a syringe‐feeding technique to minimize stress‐related biases associated with oral gavage [39]. The lactose–sucrose excipients improved solutions’ palatability, ensuring voluntary intake. The tested MIM‐seq has been developed for sublingual administration in humans. Sublingual immunotherapy is already an efficient treatment of allergic and acute disorders [40]. The oral mucosa is relatively permeable and provides access to a rich vascular supply, giving rapid absorption and acceptable bioavailability [41]. Despite the differences in oral mucosa in rodents—exhibiting a thin keratinized epithelium with low epithelial extensions—the immune network is compared with that of humans, making rodents appropriate for oral mucosa immunotherapy [42].

According to previous studies, OVX rats present higher ALP and CTX‐I levels than sham rats, markers of elevated bone turnover due to estrogen deficiency [43]. The OVX status could increase the activity of ALP in osteoblasts, as the latter is a well‐known sensitive marker for bone formation [44]. At the same time, CTX‐I is cleaved during bone resorption and is a marker of osteoclast activity [45]. In both models and at different degrees, lower plasma ALP activity and CTX‐I levels were found for MIM‐seq although significant values were only obtained for CTX‐I levels in the established OP model, suggesting reduced bone resorption. This is in line with a previous in vitro study using 3D gingiva as a periodontal disease model has shown effects on collagen metabolism, showing the ability to restore collagen deposition after an inflammatory stimulus [21].

In addition, bone turnover markers OPG and RANKL showed slight modulation in the early‐onset model, with an increased OPG/RANKL ratio, which may be a key factor affecting the osteoblast‐mediated reaction [46]. In the established model, the treatment did not show remarkable changes, indicating their lack of relevance in both OP models.

Bone quality was assessed via micro‐CT for bone microarchitecture and biomechanical testing. The biomechanical properties of the L4 vertebral body were analyzed to evaluate bone quality using a compression test. The early‐onset model showed a nonsignificant increase in the maximum load for the treatment group, while the established model showed a lower, nonsignificant value. In addition, micro‐CT analysis of trabecular femur bone microarchitecture did not reveal any significant difference between the treatment and vehicle groups in both OP models. Based on previous research, given site‐specific bone loss patterns post‐OVX [36], differential responses to MIM‐seq in the L4 vertebral body and distal femur may explain these findings.

One limitation of this study is the relatively small sample size (*n* = eight to nine per group), which may have limited the statistical power to detect subtle differences, particularly in bone microarchitectural parameters. However, the number of animals used is consistent with previous studies employing OVX‐induced OP models and following ethical principles aimed at reducing animal use. Future studies with larger cohorts are warranted to confirm and expand upon these findings.

## 5. Conclusions

This study is the first to evaluate LD MIM‐seq treatment for OP management using an OVX‐induced rat model. A key strength of this research is the use of two distinct OVX‐induced OP models, allowing for the assessment of both preventive and therapeutic effects while accounting for the temporal dynamics of bone loss.

To summarize, the results suggest a slight improvement in the maximum load of the fourth lumbar vertebral body in early OP model, along with positive effects on regulating the OPG/RANKL pathway and reducing ALP activity following MIM‐seq treatment. However, these changes did not translate into significant improvements in bone quality, as assessed by microarchitecture and mechanical testing. In the established OP model, MIM‐seq treatment significantly reduced the bone resorption marker CTX‐I, though without notable improvements in bone quality. Thus, further research is needed to validate and fully understand the clinical relevance of the observed changes after MIM‐seq treatment in managing different stages of OP.

## Author Contributions

Conceptualization, Joana Maria Ramis, Marta Monjo, and Laura Garcia‐Sureda; methodology in performing experiments, Maria Del Mar Ferrà‐Cañellas, Joana Maria Ramis, and Marta Monjo; formal analysis, Maria Del Mar Ferrà‐Cañellas, Joana Maria Ramis, Marta Monjo, and Laura Garcia‐Sureda; investigation, Maria Del Mar Ferrà‐Cañellas; data curation, Maria Del Mar Ferrà‐Cañellas; writing–original draft preparation, Maria Del Mar Ferrà‐Cañellas; writing–review and editing, Joana Maria Ramis, Marta Monjo, and Laura Garcia‐Sureda; visualization, Maria Del Mar Ferrà‐Cañellas; supervision, Joana Maria Ramis, Marta Monjo, and Laura Garcia‐Sureda; project administration, Joana Maria Ramis, Marta Monjo, and Laura Garcia‐Sureda; funding acquisition, Joana Maria Ramis, Marta Monjo, and Laura Garcia‐Sureda.

## Funding

This research was funded by the Vice Presidency and Ministry of Innovation, Research and Tourism, General Directorate of Innovation and Research, from the Balearic Government (Balearic Islands, Spain), cofunded with ERDF European Regional Development Fund (ES/01/TCAI/4_2018). The contract to Maria Del Mar Ferrà‐Cañellas was funded by the Direcció General d’Investigació, Conselleria d’Investigació, Govern Balear (FPI/040/2020) and Labo’Life España SAU.

## Disclosure

All authors have read and agreed to the published version of the manuscript.

## Conflicts of Interest

The authors declare the following potential conflicts of interest concerning the research, authorship, and publication of this article: Maria Del Mar Ferrà‐Cañellas and Laura Garcia‐Sureda work for Labo’Life España, the company that cofunded this research; it is not implied that the authors have committed any misconduct in this professional relationship. The remaining authors declare no conflicts of interest.

## Data Availability

Data supporting the findings of this study, including raw measurements and processed datasets, are available from the corresponding authors upon reasonable request.
